# Delamanid suppresses CXCL10 expression *via* regulation of JAK/STAT1 signaling and correlates with reduced inflammation in tuberculosis patients

**DOI:** 10.3389/fimmu.2022.923492

**Published:** 2022-11-08

**Authors:** Min Qiao, Shanshan Li, Jinfeng Yuan, Weicong Ren, Yuanyuan Shang, Wei Wang, Rongmei Liu, Fuzhen Zhang, Qing Li, Xiao Wu, Jie Lu, Mengqiu Gao, Yu Pang

**Affiliations:** ^1^ Department of Bacteriology and Immunology, Beijing Chest Hospital, Capital Medical University/Beijing Tuberculosis & Thoracic Tumor Research Institute, Beijing, China; ^2^ Department of Gastroenterology, Shanxi Provincial People’s Hospital, Affiliate of Shanxi Medical University, Taiyuan, Shanxi, China; ^3^ Department of Tuberculosis, Beijing Chest Hospital, Capital Medical University/Beijing Tuberculosis & Thoracic Tumor Research Institute, Beijing, China; ^4^ Beijing Key Laboratory for Pediatric Diseases of Otolaryngology, Head and Neck Surgery, Beijing Pediatric Research Institute, Beijing Children’s Hospital, Capital Medical University, National Center for Children’s Health, Beijing, China

**Keywords:** delamanid, CXCL10, JAK/STAT1, tuberculosis, inflammation

## Abstract

**Background:**

Apart from bactericidal effects, anti-tuberculosis drugs can interfere with the host’s immune system. In this study, we analyzed the role of delamanid (DLM), an inhibitor of mycolic acid synthesis of mycobacterial cell wall, on human macrophages.

**Methods:**

Based on a cohort of multidrug-resistant tuberculosis (MDR-TB) patients treated with DLM, the levels of C-reaction protein (CRP) and cytokines in the plasma were monitored using immunoturbidimetric assay and flow cytometry, respectively. We investigated the role of DLM on CXCL10 expression in U937 cell model using the following methods: cell viability assay, reverse transcription-quantitative polymerase chain reaction, enzyme linked immunosorbent assay, immunoblot, and transwell co-culture assay.

**Results:**

A total of 23 MDR-TB patients were included, comprising of 13 patients treated with optimized background therapeutic regimen (OBR) plus DLM regimen (OBR+DLM) and 10 patients treated with OBR plus placebo. DLM administration was associated with a significant reduce in circulating CRP level. Correspondingly, after treatment, the level of CXCL10 in patients treated with OBR+DLM was significantly lower than that with control. Using cell model, DLM dramatically suppressed CXCL10 expression, which majorly depended on inhibiting the JAK/STAT pathway, and impaired the migration of PBMCs.

**Conclusion:**

Our data firstly demonstrate that DLM suppresses CXCL10 expression *via* regulation of JAK2/STAT1 signaling and correlates with reduced inflammation in MDR-TB patients. DLM could be used as a potential drug for immunotherapy of patients with overactive immune response due to CXCL10.

## Introduction

Tuberculosis (TB), caused by *Mycobacterium tuberculosis* (MTB) complex, continues to pose a major public health challenge (1, 2). It remains one of leading causes for morbidity and mortality globally, with an estimated 10.0 million incident TB cases and 1.49 million TB deaths in 2020 (1). The emergence of drug-resistant TB, especially multidrug-resistant TB (MDR-TB, defined as TB resistant to rifampicin and isoniazid), seriously hampers efforts to control TB (1, 3). The World Health Organization (WHO) estimated that half of MDR-TB patients were reported in India, China and Russia (1). However, only approximately 59% of patients who initiated treatment achieved favorable outcomes (1). Therefore, there is urgent need to develop novel antimicrobial agents with enhanced efficacy against multidrug-resistant tubercle bacilli (4, 5).

Efficient control of intracellular mycobacterial growth and survival requires enhanced immune response (6, 7), but excessive inflammation is associated with substantial tissue damage (8). The balanced pro- and anti-inflammatory environment is important to provide maximum benefits to individuals infected with MTB (7). Anti-TB agents can also interfere with the immune system by modulating the functions of immune cells. For instance, isoniazid induces apoptosis of activated CD4^+^ T cells in MTB-infected murine model, and leads to decreased production of Th1 cytokine in latent TB under isoniazid preventive therapy (9). Rifampicin also acts as an immunomodulator that can reduce inflammation *via* suppressing IκBα degradation and Toll-like receptor 4 signaling (10, 11). By contrast, bedaquiline treatment triggers a series of antimicrobial defense mechanisms, including phagosome-lysosome fusion and autophagy, thus enhancing the effect of mycobacterial clearance in macrophage (12). Considering the complexity of antibiotics on host defense, it is necessary to understand how antibiotics modulate the function of immune cells, which will provide insights for host-directed anti-TB therapy.

Delamanid (DLM), a nitro-dihydro-imidazooxazole derivative, exhibits promising anti-TB activity through inhibiting mycolic acid synthesis of bacterial cell wall (13, 14). It can dramatically improve favorable treatment outcomes for individuals with MDR-TB, which has been endorsed by WHO for clinical management of rifampicin-resistant/MDR-TB patients (13, 15). Macrophages, are the primary *in vivo* cell target of MTB, which play an essential role in host defense against tubercle bacilli (16). From an immunological perspective it is questionable whether there are possible interactions between DLM and the host immune response. However, limited knowledge is available regarding this aspect. A better understanding of the impact of DLM on the host immune response is required for the development of immuno-therapeutics aiming at improving drug efficacy with minimal tissue damage.

## Materials and methods

### Ethical approval

Informed consent was obtained from each patient enrolled and the collection of peripheral blood specimens were approved by the Ethics Committee of the Beijing Chest Hospital, Capital Medical University (approval No.: YJS-2020-013). The information of all individuals involved in the study were anonymized.

### Patient enrollment

Participants for the cohort were enrolled from a multinational, randomized, double-blind, placebo-controlled clinical trial, which assess the safety, pharmacokinetic profile, and efficacy of DLM in patients with MDR-TB (13). Inclusion criteria were: (1) patients aged 18-64 years; (2) patients infected with MDR-TB confirmed by phenotypical drug susceptibility testing; (3) patients with confirmed chest CT findings of tuberculosis. Exclusion criteria were: (1) history of allergy to all nitro-imidazoles and their derivatives at any time; (2) severe comorbidities or impaired renal function or impaired hepatic function; (3) clinically significant electrocardiogram changes; (4) clinically significant metabolic, gastrointestinal, neurological, or endocrine disorder, malignancy or other abnormality.

### Detection of plasma cytokines using flow cytometry

Cytometric Bead Array (CBA) was used to measure the concentrations of cytokines in the plasma collected from MDR-TB patients enrolled, including IFN-γ (cat#561515), TNF-α (cat#561516), IL-1β (cat#561509), IL-6 (cat#561512), CXCL10 (cat#558280) and CCL2 (cat#558342). BD CBA Human Soluble Protein Master Buffer Kit and BD CBA Human Enhanced Sensitivity Master Buffer Kit were used according to manufacturer’s instructions. All antibodies and relative isotype controls were purchased from BD Biosciences (San Diego, CA, USA). Flow cytometric analysis of plasma was performed by LSRFortessa flow cytometer (BD Biosciences, San Diego, CA, USA) using FCAP Array (version 3.0).

### Cell culture

A human monocytic cell line U937 cells and a human leukemic cell line THP-1 were obtained from the American Type Culture Collection (ATCC). The cells were maintained in RPMI 1640 medium (HyClone, Waltham, USA) with 10% fetal bovine serum (FBS) (Gibco, Carlsbad, CA, USA) and incubated at 37°C in a humidified 5% CO_2_ atmosphere. In all the related experiments, U937 and THP1 cells were differentiated into adherent macrophage-like cells using 100 ng/ml Phorbol 12-myristate 13-acetate (PMA, Sigma, Darmstadt, Germany) overnight.

### Bacterial strains

The mycobacterial isolates used in this study were stored in the Tuberculosis BioBank of Beijing Chest Hospital, Capital Medical University. DLM-resistant strain of MTB (DLMr-MTB) and H37Rv were obtained by cultured from frozen stocks stored at -80°C in solid Löwenstein-Jensen media for 3 weeks. DLMr-MTB strain with growth rate (in liquid medium) similar to H37Rv was used for further experiments. Resistance to DLM of DLMr-MTB strain was confirmed by drug susceptibility test, and by sequence analysis of drug-resistant gene. The *ddn* gene, encoding deazaflavin-dependent nitroreductase required for DLM activation, was PCR-amplified using primers (forward: 5’-CACCATCATCGAGCGGATTT-3’; reverse: 5’-CAAGGGCGTGAAATGGGAT-3’) and the PCR products were sent to Beijing Ruibio BiotechCo., Ltd for sequencing.

### Determination of minimum inhibitory concentration

DLM was purchased from Biochempartner (Shanghai, China). The minimum inhibitory concentration of DLM to H37Rv and DLMr-MTB was determined using alamar blue assay as previously described (17).

### Infection of U937 cells

U937 cells were seeded at 1×10^6^ cells/well in a 12-well plate and differentiated with 100 ng/ml PMA overnight. H37Rv and DLMr-MTB were cultured from frozen stocks in solid Löwenstein-Jensen media for 3 weeks. The strains were scraped and put into grinding flask, and then added into 0.05% Tween-80 (Sigma) and vortexed for 30s to disperse. The strains were diluted with RPMI 1640 medium with 10% FBS, and the optical density (OD) value of the strain diluent was detected using a BioSpectrometer (Eppendrof, Hamburg, Germany). Cells were washed three times and were infected with H37Rv or DLMr-MTB at a multiplicity of infection (MOI) of 5 for 2 h at 37°C with 5% CO_2_. After 2 h, the media were discarded, and the cells were washed three times with 1×PBS to exclude non-internalized bacteria, and cells were incubated with the fresh RPMI 1640 medium with 10% FBS supplemented with DLM, an equal volume of DMSO was used as a control.

### Cell viability assay

Cell viability assay was performed using Cell Counting Kit-8 assays (CCK8) (Dojindo, Kumamoto, Japan) according to manufacturer’s instructions. The cells were plated at a density of 5,000 cells per well in 96-wells plates with six replicate wells per group. Cells were infected as described previously. After incubated with DLM or dimethyl sulfoxide (DMSO) for 4 h, 8 h, 24 h, 10 µl CCK8 was added into the 100 µl cell culture medium and the cells were incubated at 37°C. After 4 h, the spectrophotometric absorbance was measured at 450 nm for each sample.

### Reverse transcription-quantitative polymerase chain reaction

Total RNA was extracted from using General RNA Extraction Kit (Dongsheng Biotech, China) as manufacturer’s instructions. And mRNA was reverse transcribed to cDNA using Hifair II 1st strand cDNA synthesis supermix (Yeasen Biotech, China). Then real time quantitative PCR assays were performed using Hifair qPCR SYBR green master mix (Low Rox) (Yeasen Biotech) on ABI 7500 system (Applied Biosystems). The primers for CXCL10 (forward, 5’-CACGTGGACAAAATTGGCTTG-3’; reverse, 5’-ACCTTCTCTGCTGTTCCTCTTT-3’), IL-1β (forward, 5’-ATGATGGCTTATTACAGTGGCAA-3’; reverse, 5’-GTCGGAGATTCGTAGCTGGA-3’), IFN-γ (forward, 5’-TCGGTAACTGACTTGAATGTCCA-3’; reverse, 5’-TCGCTTCCCTGTTTTAGCTGC-3’), TNF-α (forward, 5’-GAGGCCAAGCCCTGGTATG-3’; reverse, 5’-GAGGACCTGGGAGTAGATGAG-3’), IL-6 (forward, 5’-ACTCACCTCTTCAGAACGAATTG-3’; reverse, 5’-CCATCTTTGGAAGGTTCAGGTTG-3’), IL-10 (forward, 5’-GACTTTAAGGGTTACCTGGGTTG-3’; reverse, 5’-TCACATGCGCCTTGATGTCTG-3’), CD86 (forward, 5’-CTGCTCATCTATACACGGTTACC-3’; reverse, 5’-GGAAACGTCGTACAGTTCTGTG-3’), CD40 (forward, 5’-ACTGAAACGGAATGCCTTCCT-3’; reverse, 5’-CCTCACTCGTACAGTGCCA-3’), MHC-1 (forward, 5’-GATTACATCGCCTTGAACGAGG-3’; reverse, 5’-GCAGGGTAGAAGCTCAGGG-3’), were designed by Primer-BLAST (https://www.ncbi.nlm.nih.gov/tools/primer-blast). The expression of them were analyzed by the 2^−ΔΔCT^ method and was normalized to the expression of β-actin, which primers were 5’-CATGTACGTTGCTATCCAGGC-3’ (forward) and 5’-CTCCTTAATGTCACGCACGAT-3’ (reverse).

### Enzyme linked immunosorbent assay

For measurement of CXCL10, Human CXCL10 ELISA Kit (Neobioscience, China) were used according to the manufacturer’s instructions. And OD values at 450 nm were read on a Multiskan FC microplate photometer (Biotek, USA).

### Colony-forming unit counting

Cells used for bacterial counting were lysed in PBS broth containing 0.05% sodium dodecyl sulphate for 5 min at each designated time point. Three sets of serial tenfold dilutions of the lysates from each time point were prepared in PBS, and 100 μl was plated on 7H10 agar (BD) supplemented with 10% oleic acid-albumin-dextrose-catalase (OADC). The colonies were counted after 3-4 weeks.

### Transwell co-culture assay

For transwell co-culture assay, 8 μm PC transwell (Corning, NY, USA) was used. The peripheral blood mononuclear cells (PBMC) were donated by one healthy volunteer and maintained in RPMI 1640 medium to starve for 24 h advanced. At the same time, macrophages were treated with DLM or DMSO for 24 h, then the starved PBMC were added in the upper chamber and co-cultured together with macrophage culture supernatant for another 12 h though transwell assay. Furthermore, Recombinant Human CXCL10 (GenScript, Beijing, China) and AMG487 (Selleck) were added into the lower chamber at a concentration of 1 μg/ml and 10 μM for 24 h as the positive and negative control, respectively. Inside of each insert was swabbed gently using cotton swabs and then washed with 1×PBS. Crystal violet stain solution were added to each insert and incubate for 10 minutes, then thoroughly rinse the inserts with 1×PBS until it runs clear. Using a light microscope enumerate the number of stained cells in random fields within each insert when they dry completely. The frequency of purple dots in the image indicated the migratory ability of cells.

### Western blot analysis

For immunoblot analysis, macrophages were lysed in NP-40 (Beyotime, Shanghai) supplemented with 1 mM Phenylmethanesulfonyl fluoride (PMSF) and 1% protein phosphatase inhibitor (Solarbio, Beijing). Proteins were separated by SDS-PAGE and transferred to PVDF membrane (Millipore). The blots were blocked with 5% nonfat dry milk in Tris-buffered saline with Tween-20 (TBST) for 2 h at room temperature and subsequently incubated with primary antibodies overnight at 4°C. NF-kappaB p65 (D14E12) Rabbit mAb (CST, cat#8242, 1:1000 dilution), phospho-NF-kappaB p65 (S536) Rabbit Ab (CST, cat#3031, 1:500 dilution), p38 MAPK Rabbit Ab (CST, cat#9212,1:1000 dilution), phospho-p38 MAPK (T180/T182) Rabbit Ab (CST, cat#9211,1:500 dilution), JAK2 (D2E12) Rabbit mAb (CST, cat#3230, 1:1000 dilution), phospho-JAK2 (Tyr1007) (D15E2) Rabbit mAb (CST, cat#4406,1:500 dilution), STAT1 (D1K9Y) Rabbit mAb (CST, cat#14994,1:1000 dilution), phospho-STAT1 (Ser727) (Abcam Biotechnology, cat#109461, 1:2000 dilution), Monoclonal Anti-β-Actin antibody produced in mouse (Sigma, cat#A2228, 1:5000 dilution) were used as primary antibodies. Subsequently, the membranes were incubated with goat anti-mouse IgG or goat anti-rabbit IgG (from Beijing zhongshan golden bridge biotechnology) conjugated to HRP at a dilution of 1:10,000 in blocking buffer for 2 h at room temperature. Finally, the blots were developed by Immobilon Western Chemiluminescent HRP Substrate (Solarbio, Beijing) and exposed to X-ray film. Densitometric quantification of the protein bands was performed using ImageJ 1.51j8 with a standard “gel analysis” tool according to the developer’s guidelines.

### Statistical analysis

Data shown in graphs were presented as mean ± SEM. T test, one- or two-way ANOVA analysis followed by multiple comparisons was used for statistical analysis of continuous variables, and the Fisher exact test were used for categorical variables. The quantified data with statistical analysis were performed using GraphPad Prism 8.0, Values of *P*<0.05 were considered statistically significant. All experiments were performed at least three times.

## Results

### DLM administration was associated with a significant reduce in circulating C-reaction protein level

A total of 23 MDR-TB patients were finally included in our study, 13 MDR-TB patients treated with optimized background therapeutic regimen (OBR) plus DLM regimen (OBR+DLM) and 10 MDR-TB patients treated with OBR plus placebo. The demographic and clinical characteristics of two groups were shown in **Table 1**. There were no statistically significant differences in age, gender, body-mass index (BMI), smoke, lung cavities, extent, hypertension, diabetes, adverse events and sputum culture conversion.

**Table 1:** Demographic and Baseline Clinical Characteristics.

Characteristic	OBR+placebo (n=10)	OBR+DLM (n=13)	*P* value
Age (M±SD)	36.20±9.74	39.08±11.43	0.51
Sex (male) No. (%)	6 (60.00)	10 (76.92)	0.65
BMI (M±SD)	20.81±3.26	21.11±2.75	0.81
Smoke (Yes) No. (%)	4 (40.00)	7 (53.85)	0.68
Hypertension (No) No. (%)	10 (100.00)	12 (92.31)	1.00
Diabetes (No) No. (%)	10 (100.00)	11 (84.62)	0.49
Lung cavities (Yes) No. (%)	8 (80.00)	10 (76.92)	1.00
Extent (Bilateral) No. (%)	8 (80.00)	11 (84.62)	1.00
Adverse event (Yes) No. (%)	5 (50.00)	9 (69.23)	0.42
Sputum culture conversion (Yes) No. (%)	3 (30.00)	6 (46.15)	0.67

OBR, optimized background therapeutic regimen; DLM, delamanid; BMI, body-mass index, the body-mass index is the weight in kilograms divided by the square of the height in meters.

**Figure 1:**
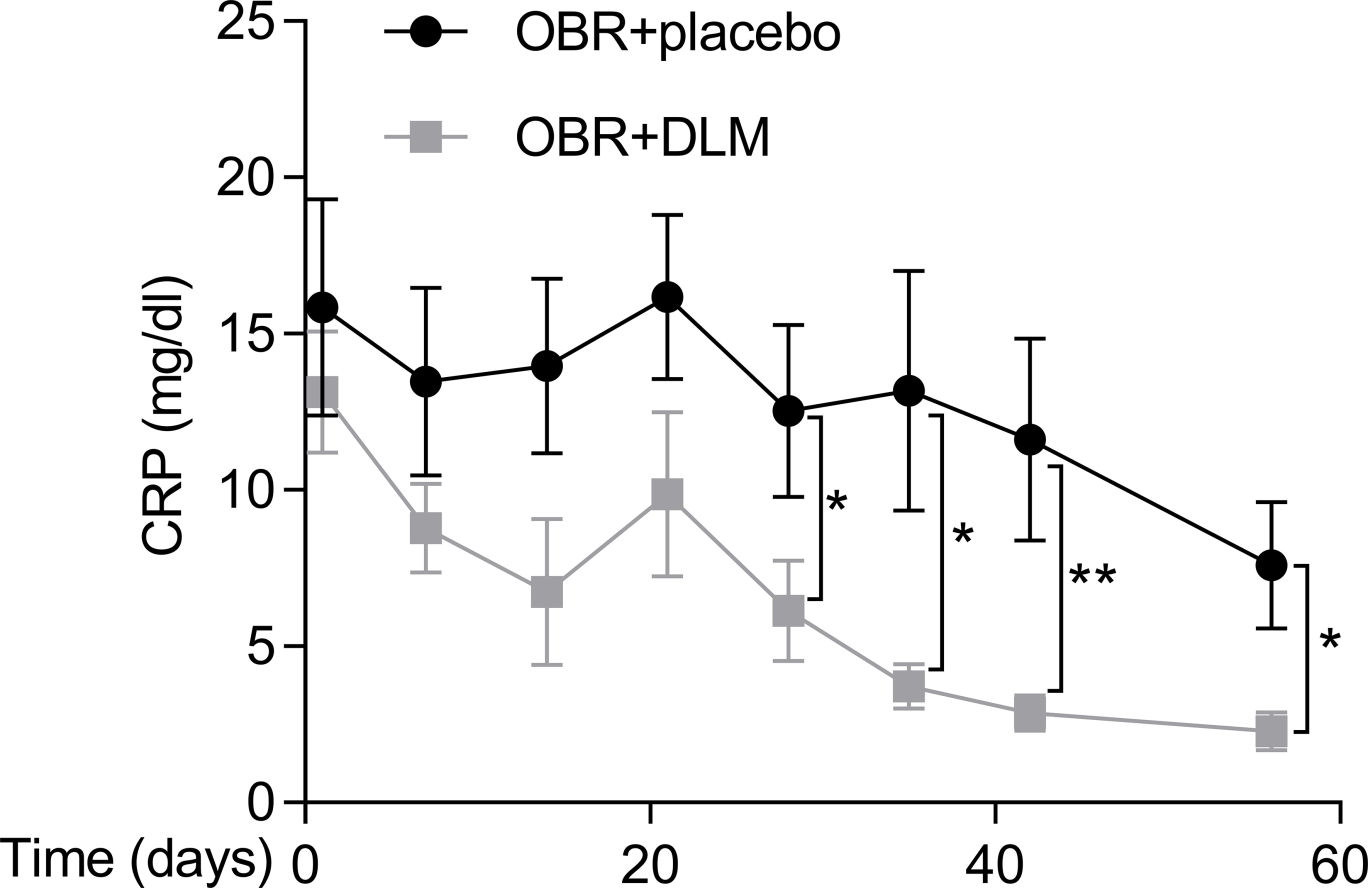
Dynamic changes in C-reactive protein level of MDR-TB patients within 8 weeks after treatment. The levels of CRP in two group patients. Data are presented as mean ± SEM. *p <.05, **p <.01 (t test).

We detected the circulating CRP levels of these patients using immunoturbidimetric assay during anti-TB treatment. As shown in **Figure 1**, the circulating CRP levels in OBR+DLM group at 28, 35, 42 and 56 days were significantly lower than those in control group (6.15 ± 6.07 at 28 days, 3.72 ± 2.70 at 35 days, 2.81 ± 2.16 at 42 days and 2.22 ± 2.30 at 56 days), respectively. This result indicated that DLM administration was associated with a significant reduce in circulating CRP level. Considering circulating levels of CRP could reflect systemic inflammation of TB patients, these results suggested that DLM might play a potential role on inhibition of inflammation.

### DLM inhibited CXCL10 expression in MDR-TB patients

**Figure 2:**
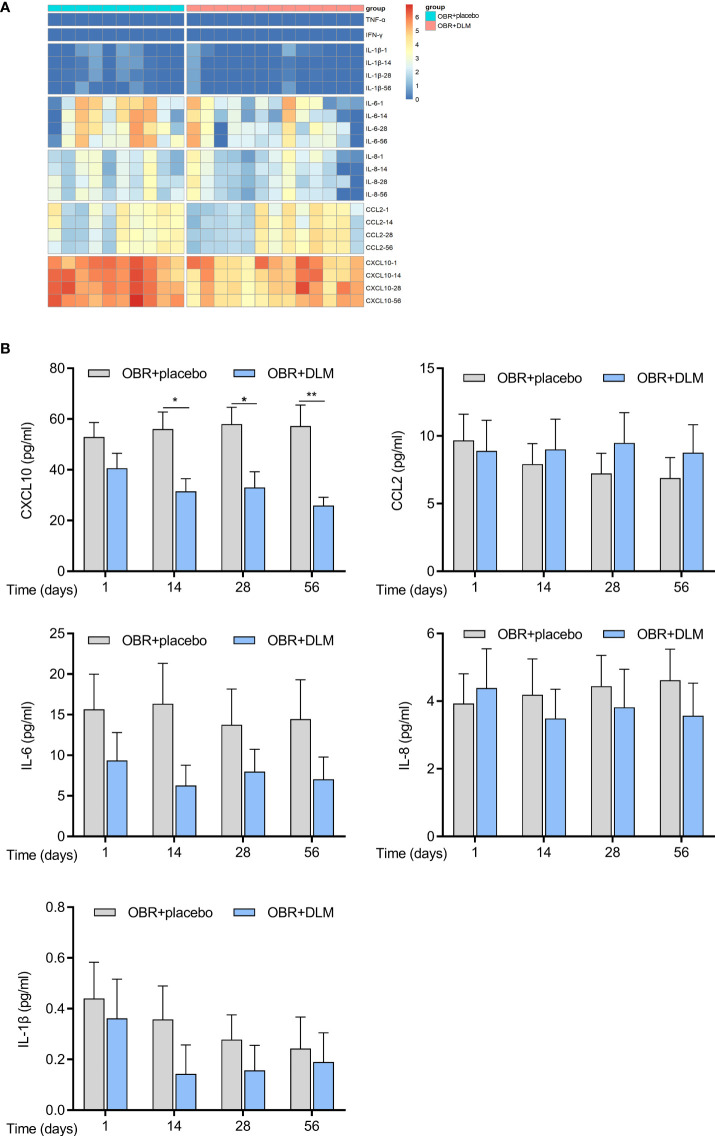
DLM modulates cytokine levels of in MDR-TB patients. Plasma from 23 individual donors were collected after treating with DLM (100 mg twice/day) or not for 1, 14, 28, 56 days. **(A)** Heatmap showing differential expression of cytokine differentially expressed by MDR-TB patients. Each column corresponds to one donor, data were normalized to determine the log ratio with respect to the median expression of each cytokine. **(B)** The levels of cytokines in two group patients. Data are presented as mean ± SEM. *p <.05, **p <.01 (two-way ANOVA). Results are representatives from at least three independent experiments.

To reflect the inflammatory levels during TB treatment, we examined the expression levels of several related cytokines and chemokines including CXCL10, CCL2, IL-6, IL-8, IL-1β, TNF-α, and IFN-γ (18–21) by flow cytometry analysis using the plasma samples from our cohort. The expression profiles of plasma cytokines were shown in **Figure 2A**, and the levels of cytokines were showed in **Figure 2B**. After treatment for 14, 28, and 56 days, the level of CXCL10 in patients treated with OBR+DLM was significantly lower than that in control group. The levels of CCL2, IL-6, IL-8 and IL-1β had no significant differences between two groups, and the levels of TNF-α and IFN-γ were under the limit of detection.

### DLM suppressed CXCL10 expression in naive and MTB-infected macrophages

**Figure 3:**
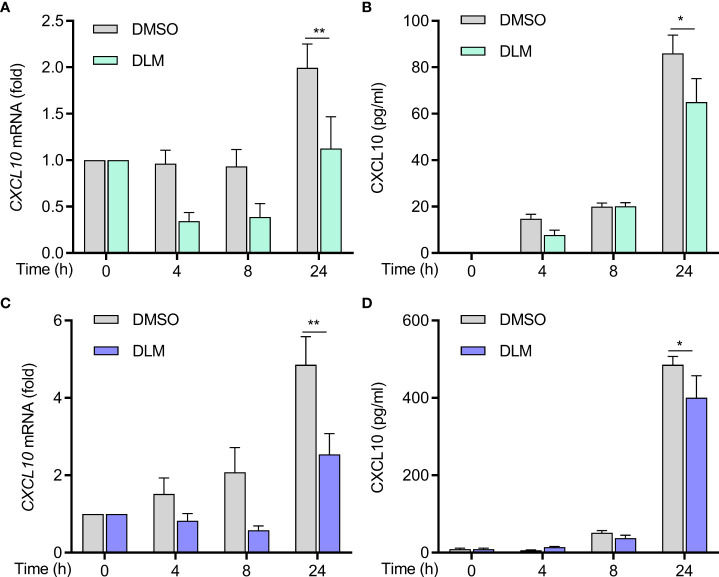
Decreased production of CXCL10 from DLM treated naive and DLMr-MTB infected macrophages. Macrophages were treated with DLM (0.3 μg/mL) for 0-24 h, DMSO was set as control group. **(A)** Quantitative PCR analysis of CXCL10 mRNA and **(B)** ELISA of CXCL10 in supernatants of cells from each group. Macrophages were infected with DLMr-MTB and then incubated for an additional 0-24 h with DLM (0.3 μg/mL). DLM treatment led to a decrease in CXCL10 in infected macrophages **(C, D)**. Data are presented as mean ± SEM. *p<.05, **p<.01 (two-way ANOVA). Results are representatives from at least three independent experiments.

To explore whether DLM could influence CXCL10 expression in macrophage, U937 cells were differentiated into adherent macrophage-like cells. We treated macrophages with DLM at 0.3 μg/mL, which corresponds to the concentration detected in the plasma of TB patients treated with DLM (22), using DMSO-treated cells as control. By performing RT-qPCR analysis, the results showed that the mRNA level of CXCL10 in macrophages treated with DLM was significantly lower than that of control group (**Figure 3A**). Consistently, lower production of CXCL10 from macrophages treated with DLM was observed *via* ELISA, compared to control group (**Figure 3B**). And the cell viability of macrophage was not affected by DLM (**Figure S1A**).

We next evaluated if DLM could suppress CXCL10 expression in MTB-infected cells. We generated a DLM-resistant strain of M. tuberculosis (DLMr-MTB) to exclude potential differences caused by the MTB bacillary load between treated and untreated cells. The DLMr-MTB strain carried a base 134 deletion mutation in ddn, the enzyme catalyzing the reduction of DLM to release reactive nitrogen species (**Figure S2A**). As shown in **Figure S2B**, the DLMr-MTB strain had a similar generation time to H37Rv when cultured in 7H9 liquid medium, and DLM (0.3 μg/ml) could inhibit the growth of H37Rv but not DLMr-MTB strain. The MIC of the DLMr-MTB strain was higher than 32 mg/L, and the MIC of H37Rv was lower than 0.03 mg/L, similar to previously published study (23). Macrophages were infected with DLMr-MTB for 2 h and then incubated for an additional 0-24 h with DLM (0.3 μg/mL) and DMSO, respectively. Compared to DMSO, DLM treatment led to a decrease in CXCL10 expression in MTB-infected macrophages as well (**Figures 3C, D**). And the cell viability of macrophage infected with DLMr-MTB was not affected by DLM (**Figure S1B**). We also found that the mRNA level of IL-1β, IFN-γ, TNF-α, IL-6, and CD 86 in U937 derived macrophages treated with DLM was significantly lower than that of control group no matter the cells were infected by DLMr-MTB or not (**Figures S3** and **S4**), and these results were further validated using THP1 cells treated with DLM or DMSO (**Figure S5**), which indicated that DLM could influence macrophages activation and polarization.

### DLM did not influence macrophage bactericidal functions

To explore whether DLM could influence macrophage bactericidal functions, we treated MTB-infected macrophages with DLM and performed CFU assay. Compared to DMSO, DLM treatment after 8 h and 24 h led to significant decreases in the bacillary load of H37Rv-infected macrophages (**Figure S6A**). And the cell viability of these macrophages weas not affected (**Figure S1C**). In contrast, there was no statistically significant difference in the bacillary load of DLMr-MTB inside macrophages between DLM and DMSO groups (**Figure S6B**). These results revealed that DLM could inhibit the MTB growth in macrophages only by bactericidal function of the drug itself but not by modulating bactericidal function of macrophage.

### DLM inhibited the migration of PBMC by suppressing CXCL10 expression

**Figure 4:**
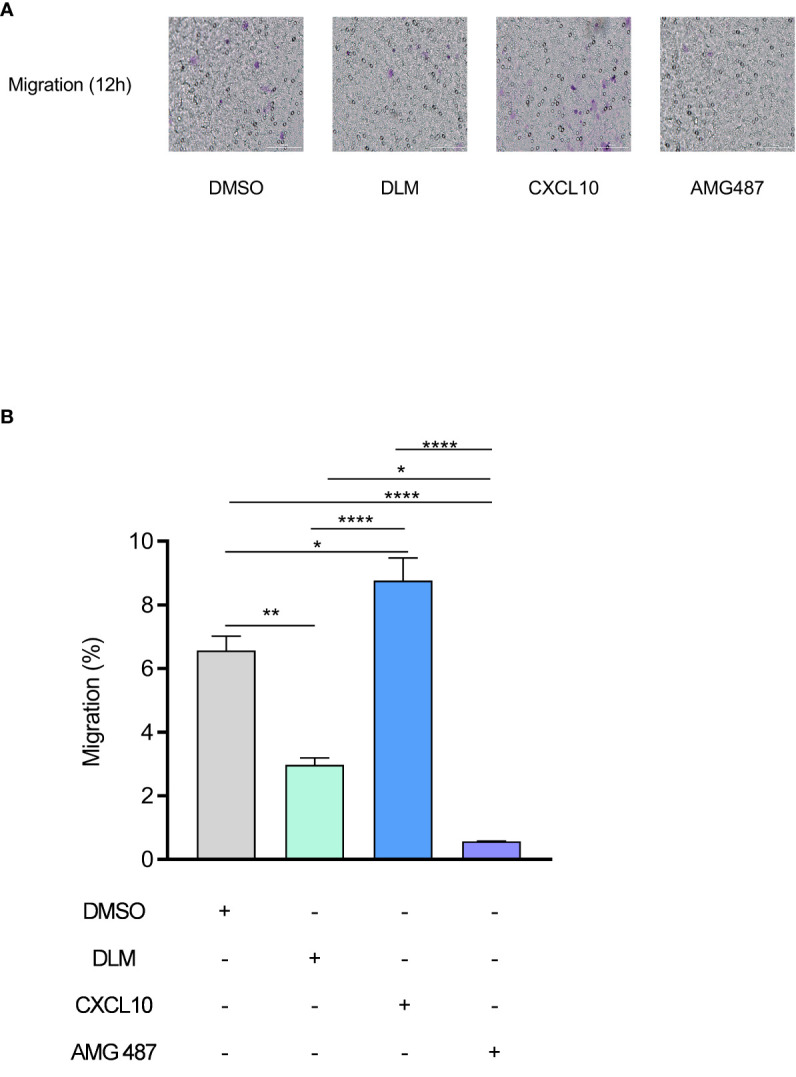
DLM inhibits the migration of PBMC by suppressing CXCL10 expression. Macrophages were pretreated with DMSO, DLM, CXCL10 and AMG487 for 24 h respectively to compare the influence on migratory ability of PBMC. The purple dots in the image indicate the migrated cells. Number of migrated PBMC by group (objective ×20) **(A)**. PBMC migration rate of each group **(B)**. Data are presented as mean ± SEM. *p<.05, **p<.01, and ****p <.0001 (two-way ANOVA). Results are representatives from at least three independent experiments.

CXCL10 plays an important role during infections by stimulating the migration of immune cells to the infected sites. To determine whether DLM has a potential to inhibit the migration of immune cells, we treated PBMCs with DMSO, DLM, CXCL10 and AMG487 (a CXCR3 small molecule inhibitor), respectively. Transwell migration assay revealed that recombinant Human CXCL10 remarkably promoted the migration of PBMCs (**Figure 4**, p=0.030). However, DLM and AMG487 significantly inhibited the migration of PBMCs (**Figure 4**).

### DLM inhibited CXCL10 expression in macrophages *via* the JAK2/STAT1 pathway

**Figure 5:**
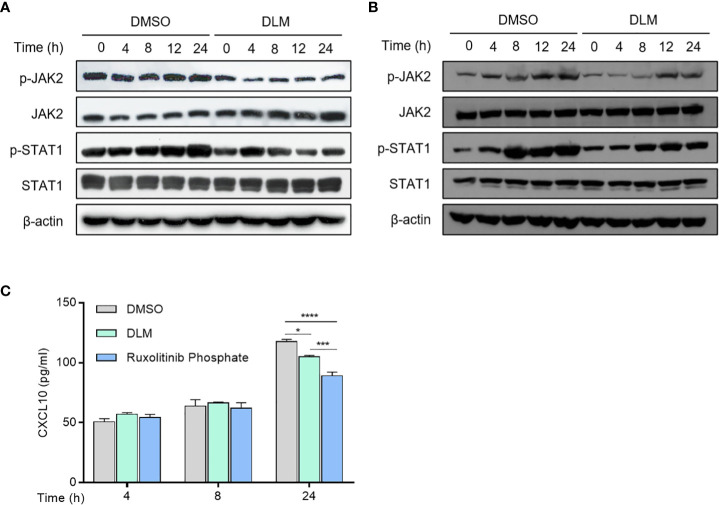
Effect of DLM on JAK/STAT1 pathway in macrophages. **(A)** Macrophages were treated with DLM or DMSO, respectively, for 0-24 h. **(B)** Macrophages were infected with DLMr-MTB and then incubated for an additional 0-24 h with DLM (0.3 μg/mL). Western blot analysis was performed by using antibodies to p-JAK2, JAK2, p-STAT1 and STAT1, respectively. β-actin was used as control. **(C)** Macrophages were treated with DLM, DMSO, Ruxolitinib phosphate (1uM), respectively, for 0-24 h. The cell culture supernates were subjected to ELISA analysis to measure CXCL10 protein level. Data are presented as mean ± SEM. *p<.05, ***p<.001, and ****p < .0001 (two-way ANOVA). Results are representatives from at least three independent experiments.

Multiple signaling pathways participate in regulating the transcription of CXCL10, including NF-κB, MAPK and JAK-STAT pathways (24–26), we thus evaluated the effects of DLM on these pathways in macrophages. Western blot analysis showed DLM treatment led to a reduction in JAK2 and STAT1 phosphorylation in a time-dependent manner (**Figure 5A**; **Figure S8**), whereas the amount of total JAK2 and STAT1 proteins were not affected. Similarly, the phosphorylation of JAK2 and STAT1 in DLMr-MTB infected U937 cells treated with DLM or DMSO were reduced in accordance (**Figure 5B**). In contract, DLM treatment did not affect phosphorylation of p65 and p38 (**Figure S7**). These results indicated DLM possibly inhibits CXCL10 expression in macrophages *via* the JAK/STAT pathway, not NF-κB or MAPK pathway.

Previous reports showed that the selective pharmacological JAK2 inhibitor Ruxolitinib phosphate could significantly inhibit upregulated chemokine expression in human mesangial cells (27). Therefore, we compared the effects of DLM and Ruxolitinib phosphate on CXCL10 expression. ELISA analysis showed that Ruxolitinib phosphate proved more effective in suppressing CXCL10 expression than DLM at 24 h (**Figure 5C**). These results suggested that DLM inhibited CXCL10 expression in U937 cells through suppressing activation of JAK2/STAT1 signaling pathway.

## Discussion

The spread of drug-resistant bacteria poses a substantial threat to morbidity and mortality worldwide, emphasizing the urgent need to develop new antibiotics (3). These agents, beyond their bactericidal activity, may alter the function of host immune cell. We describe for the first time the potential mechanisms by which DLM correlates with reduced inflammation in pulmonary TB patients. The administration of DLM significantly suppressed CXCL10 expression of host immune cells. CXCL10, a member of non-ELR (Glu-Leu-Arg) CXC chemokine, induces migration/homing of multiple immune cells to areas of inflammation by binding its unique receptor CXCR3 (28, 29). It plays an important role in the innate immune responses, and is also crucial for subsequent direction of adaptive immune responses. Previous studies showed that the CXCL10 expression levels were positively correlated with the extent of tissue injury and pathogen burden (30, 31). In COVID-19 patients, SARS-CoV-2 infection triggered excessive production of CXCL10, thereby resulting in over recruitment of inflammatory neutrophils, macrophages and Th1 lymphocytes into lung tissue which could cause pulmonary inflammation and destruction (32, 33). In our cohort, we also found that the patients treated with DLM-containing regimens showed a controlled inflammatory response, as demonstrated in lower C-reactive protein level. In line with our results, a cohort analysis by Kumar and colleagues revealed that the elevated levels of CXCL10 were associated with poor TB treatment outcomes, and could be a predictive marker for clinical outcomes (34, 35). These observations taken together indicate that DLM, in addition to direct bacterial killing, could bring treatment benefits to patients afflicted with drug-resistant tubercle bacilli *via* suppressing the secretion of CXCL10 and minimizing host inflammation.

Despite the fact that a variety of cells could secrete CXCL10 *in vivo*, analysis of human single-cell RNA-seq datasets revealed that macrophages were the predominant cell type responsible for the production of CXCL10 (36). Our results showed that DLM could inhibit CXCL10 expression in macrophages regardless of MTB infection status, which explained decreased level of CXCL10 in peripheral blood of patients initiated with multidrug treatment containing DLM.

**Figure 6:**
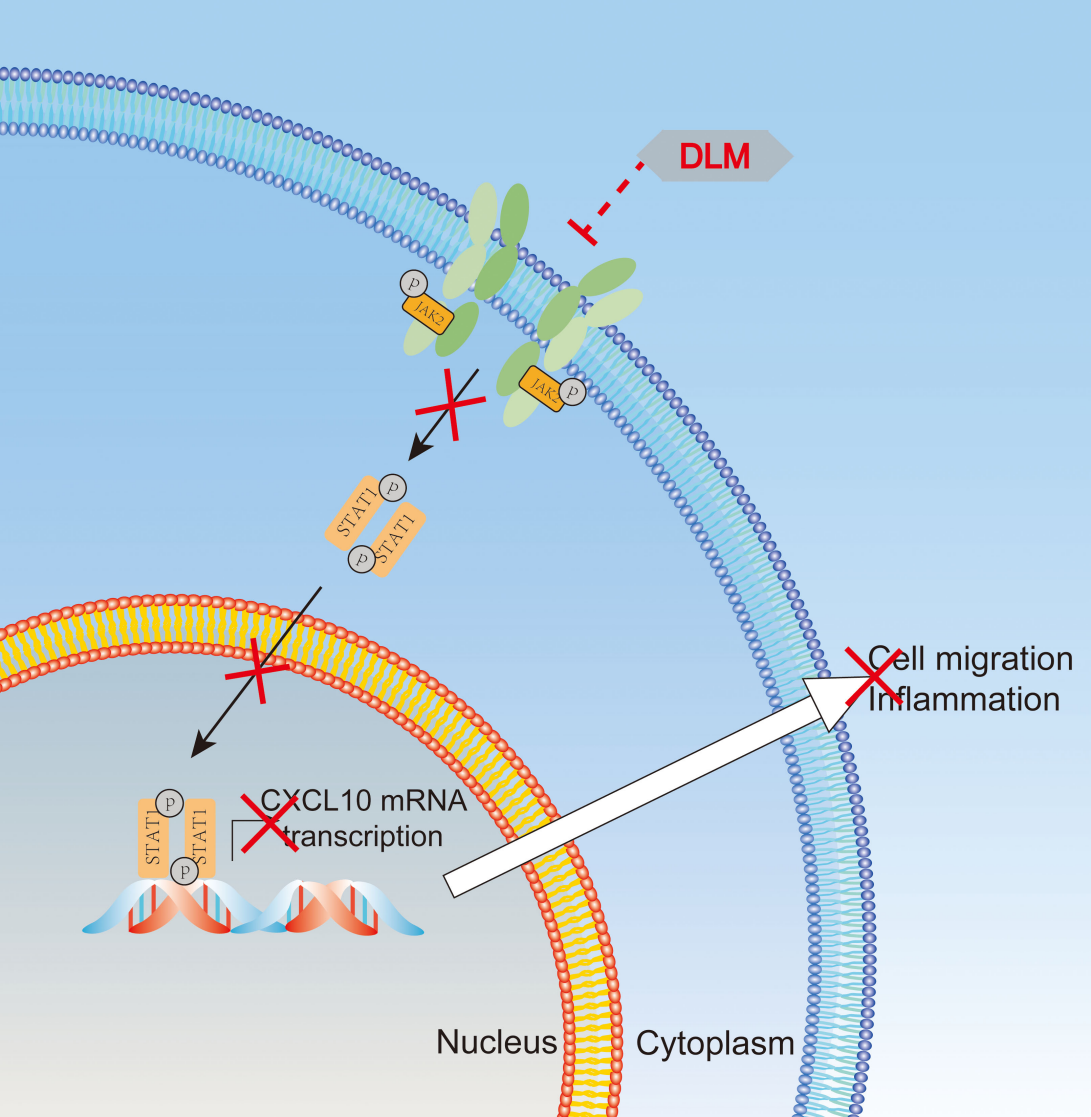
Schematic representation of putative interaction of DLM with JAK2/STAT1 signaling pathways. Schema illustrates the mechanism that DLM inhibits cell migration by suppressing the JAK2/STAT1 signaling pathway. DLM inhibited the phosphorylation (Tyr1007) and activation of JAK2 and then further inhibited the phosphorylation (Ser727) and activation of STAT1. These further induced the decrease of the downstream genes of STAT1, such as CXCL10, which participated in inflammation by promoting cell migration and ultimately resulted in the progression of TB.

That macrophage secretes CXCL10 is not only of interest with respect to this study, but raises an important question about the molecular mechanisms of inhibition of CXCL10 expression in target cells by DLM. Multiple signaling pathways participate in regulating the transcription of CXCL10, including NF-κB, MAPK and JAK/STAT pathways (24–26). Using a macrophage model, our experimental data revealed that DLM suppressed phosphorylation of JAK2/STAT1 leaded to reduced CXCL10 production as shown in **Figure 6**. Recent work has highlighted the role of IFN-γ and TNF-α could induce the secretion of CXCL10 in macrophages (37). However, IFN-γ and TNF-α in plasma samples of our cohorts were under the limits of detection using cytometric bead array, and we also detected the expression levels of IFN-γ using ELISA. The results showed that there were no statistical differences between the two groups and lots of samples were also under the limits of detection (data were not shown there). As IFN-γ and TNF-α is primarily produced by cells of the immune system, including innate-like lymphocyte populations, and adaptive immune cells, and NF-kB pathway plays a major role in regulation of CXCL10 expression in monocytes stimulated by these proinflammatory cytokines (37), but not JAK2/STAT1 pathway noted in our observations. Thus, we speculate that DLM employs a distinct signaling pathway from IFN-γ and TNF-α to mediate CXCL10 production. In agreement with this hypothesis, no significant difference was observed in the serum levels of IFN-γ and TNF-α between control and DLM group. Further studies are needed to elucidate the mechanism by which DLM regulate phosphorylation status of JAK2 and STAT1 proteins.

Conventionally, much attention has been focused on identifying the agents that could boost proinflammatory responses aiming to kill and sequester the pathogens invading host cells (16, 38). However, our data showed that the down-regulated secretion of proinflammatory cytokine CXCL10 would produce additional benefit for MDR-TB patients *via* decreasing immune mediated pathology. The balance between the timing and expression levels of pro- and anti-inflammatory responses plays a key role in the fate of infection. The immune surveillance of TB patients is of great importance to identify the individuals at high risk for failure to control bacterial infection or excessive tissue damage.

We also acknowledge several obvious limitations to this study. First, the sample size is small, the conclusions drawn may be limited, and the plasma sample is frozen for a long time, and the protein degradation may affect the results. Second, we did not explore the effect of DLM on the bactericidal mechanism of macrophages in detail. Further results of these experiments are urgently needed to determine its clinical application in treating MDR-TB. Nevertheless, our data provide new insights into DLM in the clinical management of TB patients.

In conclusion, our data firstly demonstrate that DLM suppresses CXCL10 expression *via* regulation of JAK2/STAT1 signaling and correlates with reduced inflammation in TB patients. Our work highlights the importance of the balance between pro- and anti-inflammatory responses against MTB infections. In addition, our data also indicates that DLM could be used as a potential drug for immunotherapy of patients with overactive immune response due to CXCL10. Further studies are needed to elucidate the mechanism by which DLM regulate phosphorylation status of JAK2 and STAT1 proteins.

## Data availability statement

The original contributions presented in the study are included in the article/**Supplementary Material**. Further inquiries can be directed to the corresponding authors.

## Ethics statement

The studies involving human participants were reviewed and approved by Ethics Committee of the Beijing Chest Hospital, Capital Medical University. The patients/participants provided their written informed consent to participate in this study.

## Author contributions

JL, MG, and YP conceived and designed the work; SL and WR collected the samples; MQ, JY, WW, RL, and FZ performed the experiments; YS, QL, and XW analyzed the data; JL, YP, and MQ prepared the manuscript. All authors contributed to the article and approved the submitted version.

## Funding

This work was supported by the Beijing Science and Technology Planning Project (Z191100006619077), and the Capital’s Funds for Health Improvement and Research (2020-1-1041).

## Acknowledgments

The authors acknowledge the staff at Beijing Chest Hospital for their cooperation and support.

## Conflict of interest

The authors declare that the research was conducted in the absence of any commercial or financial relationships that could be construed as a potential conflict of interest.

## Publisher’s note

All claims expressed in this article are solely those of the authors and do not necessarily represent those of their affiliated organizations, or those of the publisher, the editors and the reviewers. Any product that may be evaluated in this article, or claim that may be made by its manufacturer, is not guaranteed or endorsed by the publisher.

## Supplementary material

Supplementary Figure 1 Cell viability assay of macrophages incubated with DLM. Cells were treated with DLM (0.3μg/ml) **(A)**, Cells were infected with DLMr-MTB **(B)**, or H37Rv **(C)** for 2 h and then treated with DLM (0.3 μg/mL) for an additional 0-24 h. Cell viability was evaluated with the CCK8 assay according to the manufacturer’s instructions. Results represent the mean ± SEM of six replicates. One representative experiment (out of three) is shown.

Supplementary Figure 2 Generation of DLMr-MTB. **(A)** Amino acid sequence alignment of the Ddn in H37Rv and DLMr-MTB strain. The deletion mutation was at position 45. **(B)** Growth of H37Rv and DLMr-MTB in 7H9 medium supplemented with 10% OADC in enrichment with DLM or not. Optical density (OD) measurements of bacterial growth of H37Rv and DLMr-MTB. Data are presented as mean ± SEM. Results are representatives from at least three independent experiments.

Supplementary Figure 3 Effect of DLM on macrophages activation, polarization and antigen-presentation. Macrophages were treated with DLM (0.3 μg/mL) for 0-24 h, DMSO was set as control group. Quantitative PCR analysis of **(A)** IL-1β, **(B)** IFN-γ, **(C)** TNF-α, **(D)** IL-6, **(E)** IL-10, **(F)** CD 86, **(G)** CD 40 and **(H)** MHC-1 mRNA to reflect macrophages activation, polarization and presentation. Data are presented as mean ± SEM. **p*<.05, ***p*<.01, ***p<.001, *****p* <.0001 (two-way ANOVA). Results are representatives from at least three independent experiments.

Supplementary Figure 4 Effect of DLM on DLMr-MTB infected macrophages activation, polarization and antigen-presentation. Macrophages were infected with DLMr-MTB and then incubated for an additional 0-24 h with DLM (0.3 μg/mL). Quantitative PCR analysis of **(A)** IL-1β, **(B)** IFN-γ, **(C)** TNF-α, **(D)** IL-6, **(E)** IL-10, **(F)** CD 86, **(G)** CD 40 and **(H)** MHC-1 mRNA to reflect macrophages activation, polarization and presentation. Data are presented as mean ± SEM. ***p*<.01, ***p<.001, *****p* <.0001 (two-way ANOVA). Results are representatives from at least three independent experiments.

Supplementary Figure 5 Effect of DLM on THP-1 cells activation, polarization and antigen-presentation. THP-1 cells were treated with DLM (0.3 μg/mL) for 0-24 h, DMSO was set as control group. Quantitative PCR analysis of **(A)** IL-1β, **(B)** IFN-γ, **(C)** TNF-α, **(D)** IL-6, **(E)** IL-10, **(F)** CD 86, **(G)** CD 40 and **(H)** MHC-1 mRNA to reflect macrophages activation, polarization and presentation. Data are presented as mean ± SEM. **p*<.05, ***p*<.01, ***p<.001, *****p* <.0001 (two-way ANOVA). Results are representatives from at least three independent experiments.

Supplementary Figure 6 DLM did not activate macrophage bactericidal functions. Macrophages were infected with H37Rv **(A)** or DLMr-MTB **(B)** 2 h and then incubated with DLM for 0-24 h. At each designated time point, macrophages were lysed, and several sets of serially gradient dilution of the lysates were prepared in 7H9 broth and were then cultivated on 7H10 agar plates for bacterial CFU counting. Data are presented as mean ± SEM. *****p* <.0001 (two-way ANOVA). One representative experiment of three is shown.

Supplementary Figure 7 Effect of DLM on NF-κB and MAPK pathway in macrophages. U937 cells were treated with DLM or DMSO, respectively, for 0-24 h. Western blot analysis was performed by using antibodies to p-p65, p65, p-p38, and p38, respectively. β-actin was used as control.

Supplementary Figure 8 Effect of DLM on JAK/STAT1 pathway in THP-1 cells. THP-1 cells were treated with DLM, DMSO, respectively, for 0-24 h. Western blot analysis was performed by using antibodies to p-JAK2, JAK2, p-STAT1, STAT1, respectively. β-actin was used as control.
