# Supplementary figures and images for: Delamanid suppresses CXCL10 expression via regulation of JAK/STAT1 signaling and correlates with reduced inflammation in tuberculosis patients
Source: Front Immunol. 2022 Nov 8;13:923492. doi: 10.3389/fimmu.2022.923492 (PMC9679411; doi:10.3389/fimmu.2022.923492)

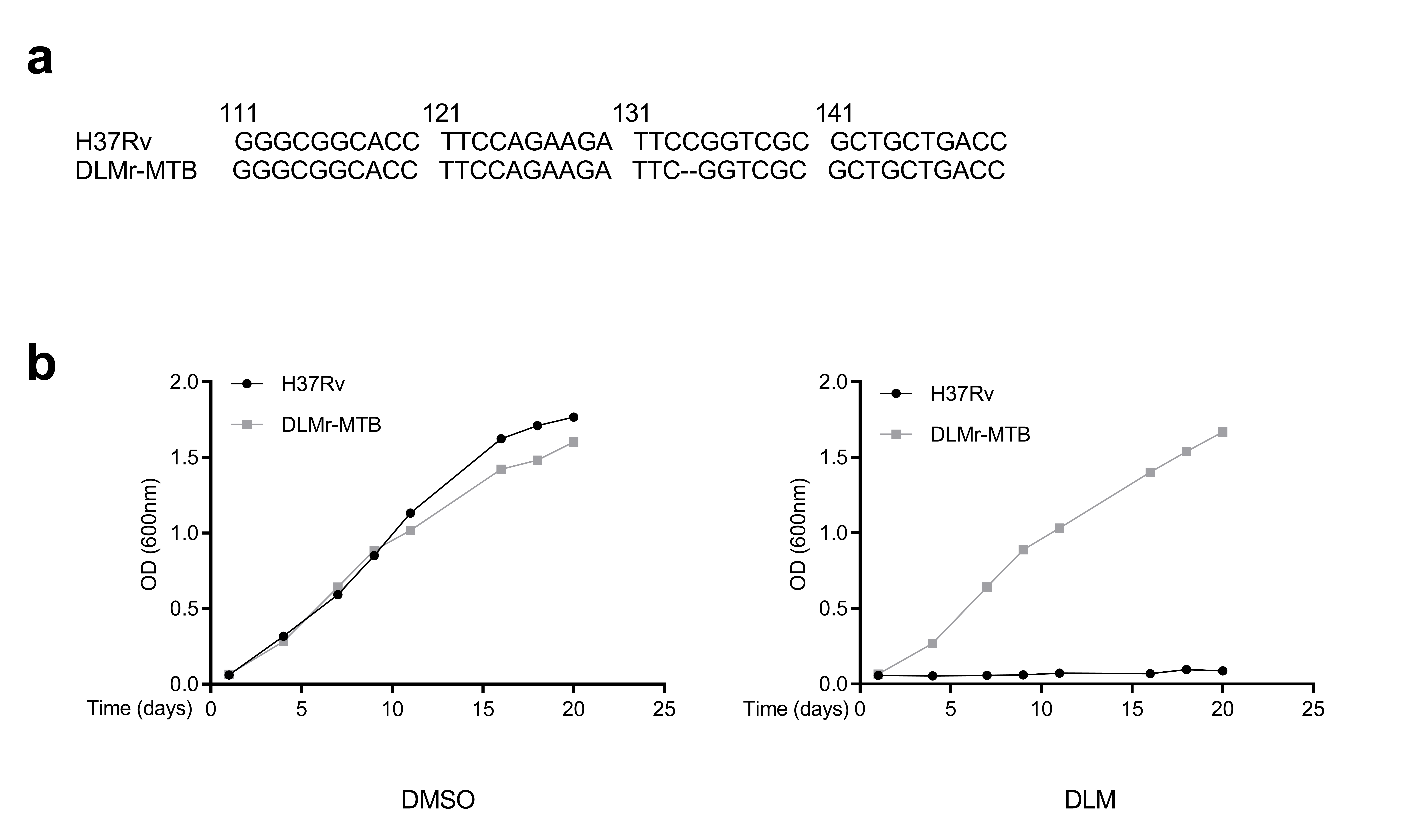

Supplement: Supplementary Figure 2 — Generation of DLMr-MTB. (A) Amino acid sequence alignment of the Ddn in H37Rv and DLMr-MTB strain. The deletion mutation was at position 45. (B) Growth of H37Rv and DLMr-MTB in 7H9 medium supplemented with 10% OADC in enrichment with DLM or not. Optical density (OD) measurements of bacterial growth of H37Rv and DLMr-MTB. Data are presented as mean ± SEM. Results are representatives from at least three independent experiments. [file Image_2.tif]

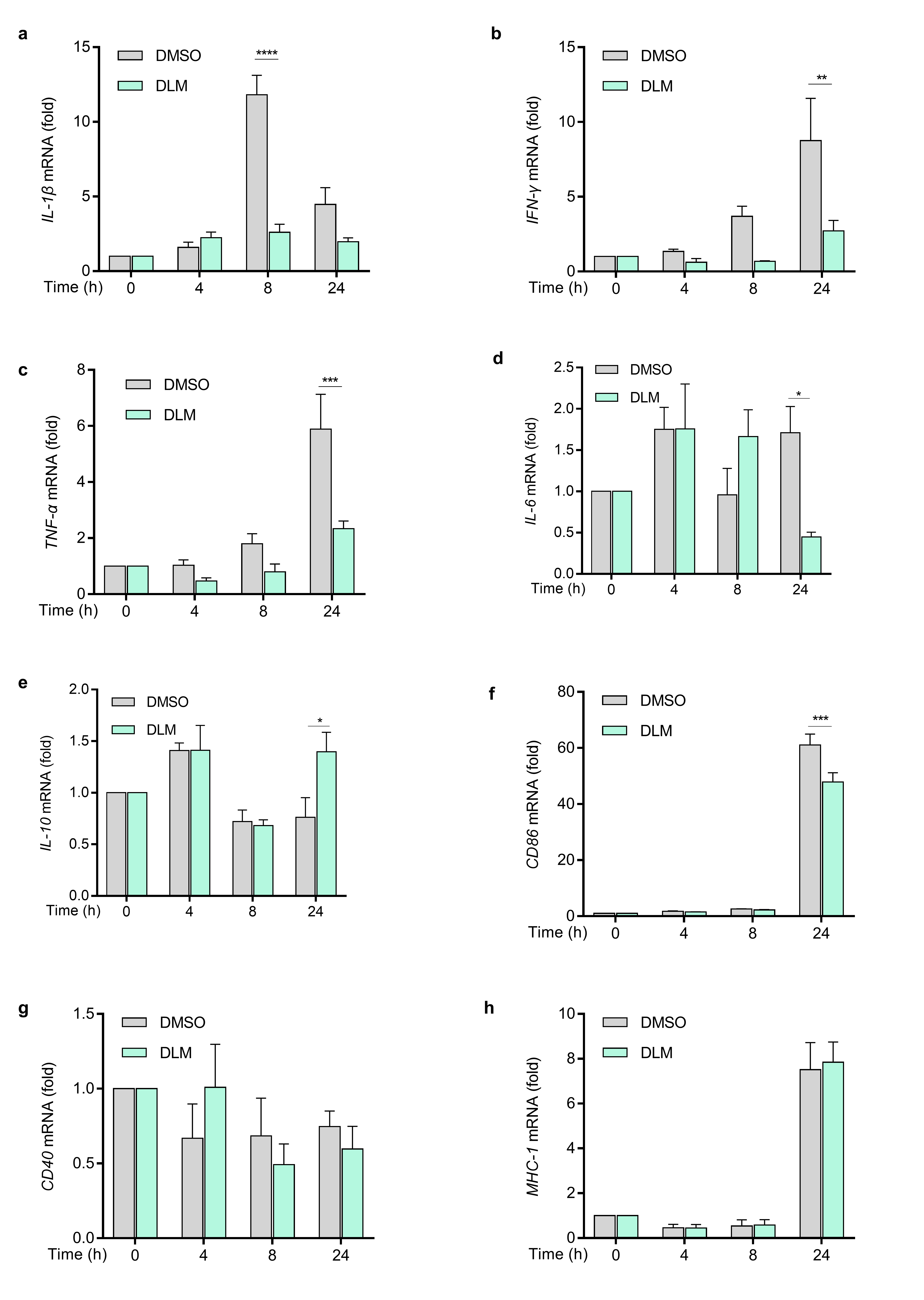

Supplement: Supplementary Figure 3 — Effect of DLM on macrophages activation, polarization and antigen-presentation. Macrophages were treated with DLM (0.3 μg/mL) for 0-24 h, DMSO was set as control group. Quantitative PCR analysis of (A) IL-1β, (B) IFN-γ, (C) TNF-α, (D) IL-6, (E) IL-10, (F) CD 86, (G) CD 40 and (H) MHC-1 mRNA to reflect macrophages activation, polarization and presentation. Data are presented as mean ± SEM. *p<.05, **p<.01, ***p<.001, ****p <.0001 (two-way ANOVA). Results are representatives from at least three independent experiments. [file Image_3.tif]

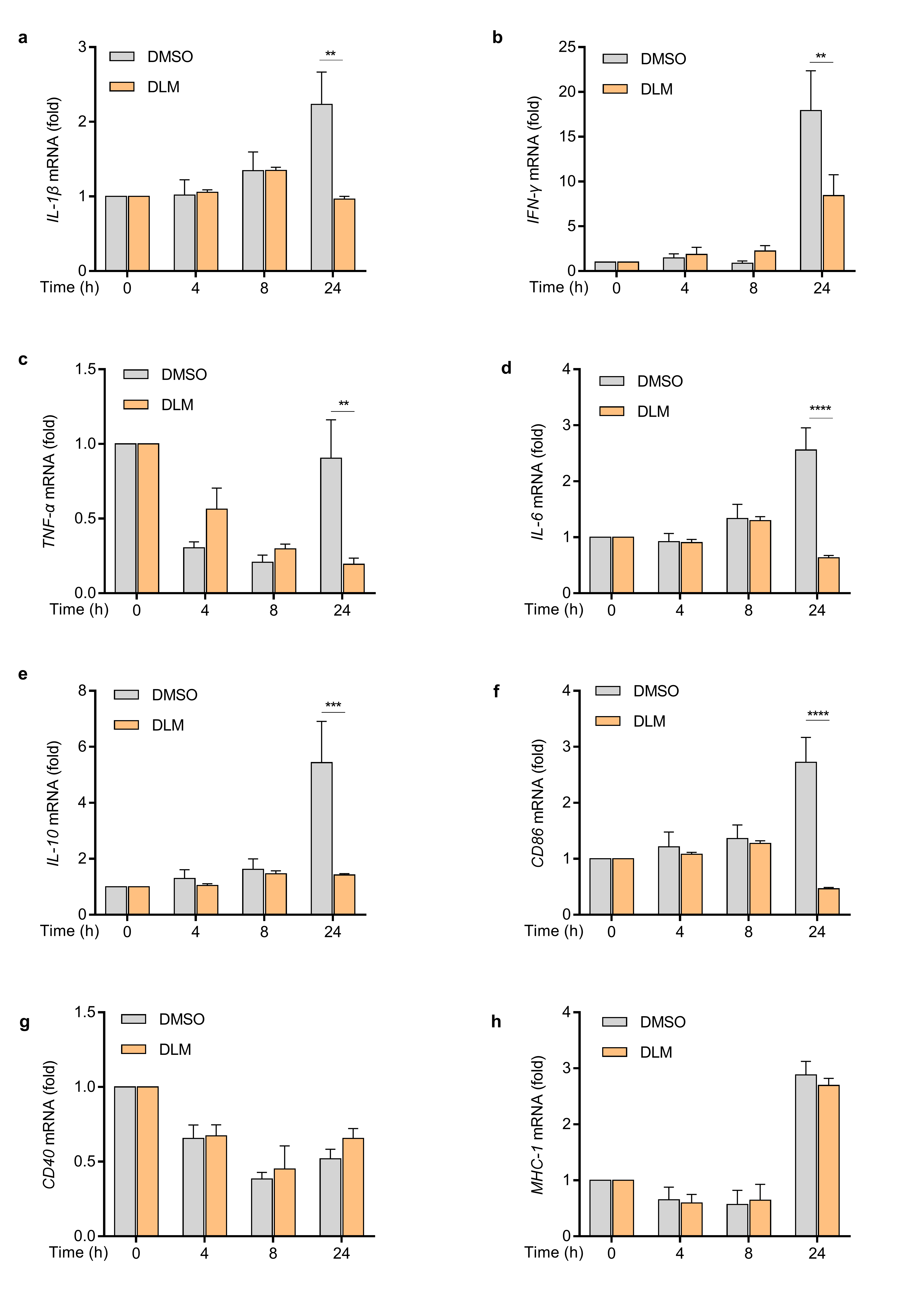

Supplement: Supplementary Figure 4 — Effect of DLM on DLMr-MTB infected macrophages activation, polarization and antigen-presentation. Macrophages were infected with DLMr-MTB and then incubated for an additional 0-24 h with DLM (0.3 μg/mL). Quantitative PCR analysis of (A) IL-1β, (B) IFN-γ, (C) TNF-α, (D) IL-6, (E) IL-10, (F) CD 86, (G) CD 40 and (H) MHC-1 mRNA to reflect macrophages activation, polarization and presentation. Data are presented as mean ± SEM. **p<.01, ***p<.001, ****p <.0001 (two-way ANOVA). Results are representatives from at least three independent experiments. [file Image_4.tif]

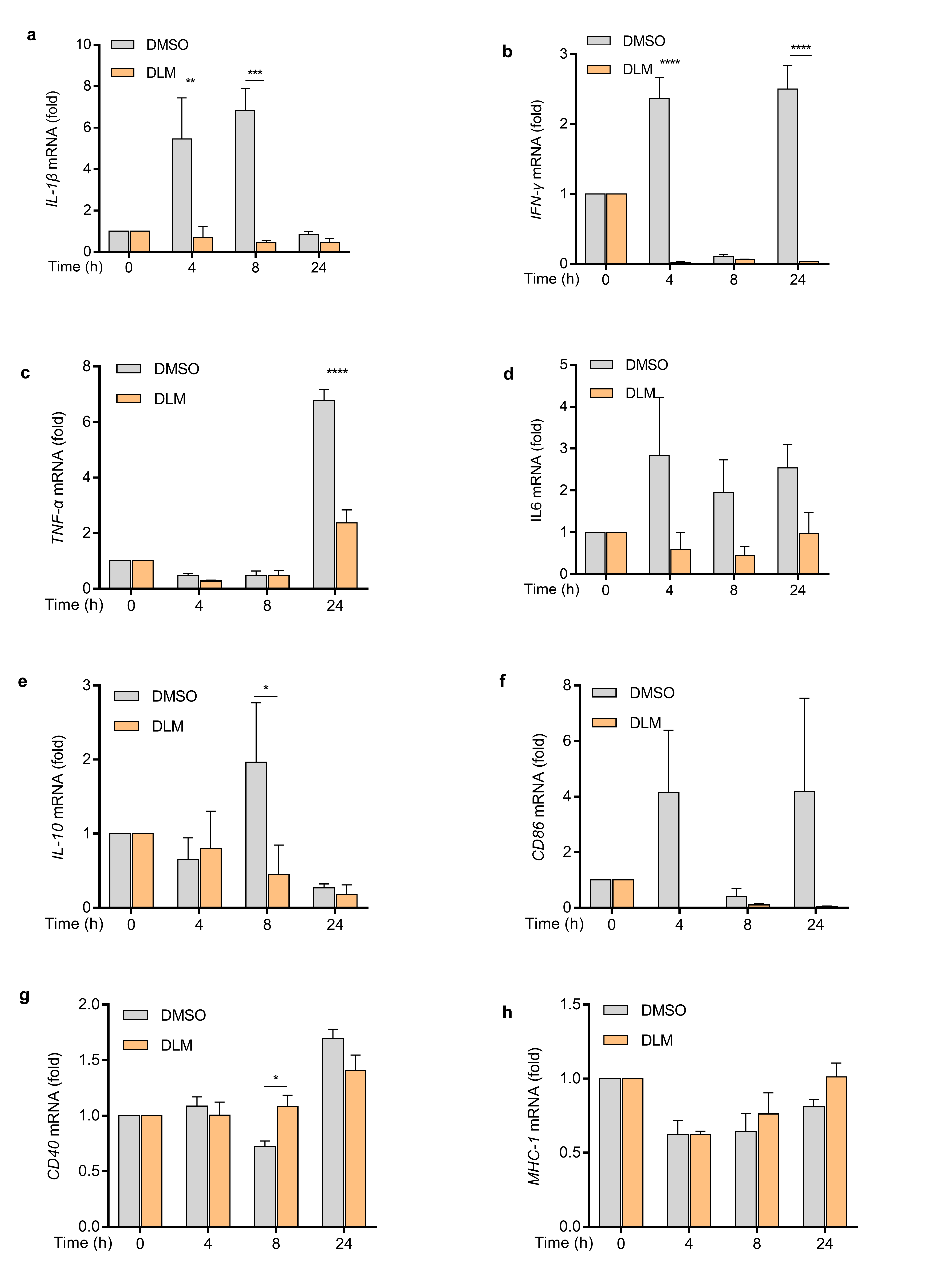

Supplement: Supplementary Figure 5 — Effect of DLM on THP-1 cells activation, polarization and antigen-presentation. THP-1 cells were treated with DLM (0.3 μg/mL) for 0-24 h, DMSO was set as control group. Quantitative PCR analysis of (A) IL-1β, (B) IFN-γ, (C) TNF-α, (D) IL-6, (E) IL-10, (F) CD 86, (G) CD 40 and (H) MHC-1 mRNA to reflect macrophages activation, polarization and presentation. Data are presented as mean ± SEM. *p<.05, **p<.01, ***p<.001, ****p <.0001 (two-way ANOVA). Results are representatives from at least three independent experiments. [file Image_5.tif]

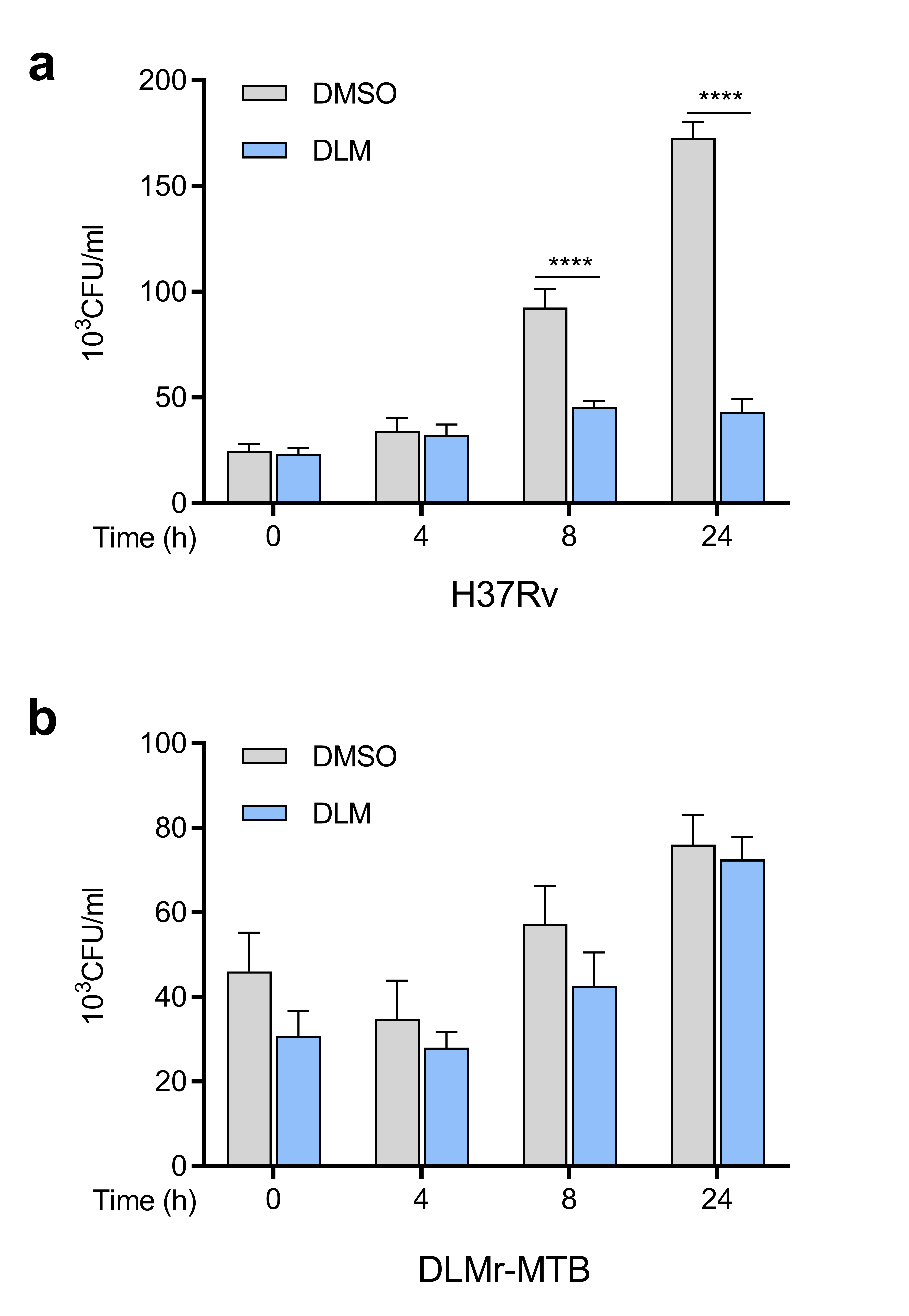

Supplement: Supplementary Figure 6 — DLM did not activate macrophage bactericidal functions. Macrophages were infected with H37Rv (A) or DLMr-MTB (B) 2 h and then incubated with DLM for 0-24 h. At each designated time point, macrophages were lysed, and several sets of serially gradient dilution of the lysates were prepared in 7H9 broth and were then cultivated on 7H10 agar plates for bacterial CFU counting. Data are presented as mean ± SEM. ****p <.0001 (two-way ANOVA). One representative experiment of three is shown. [file Image_6.tif]

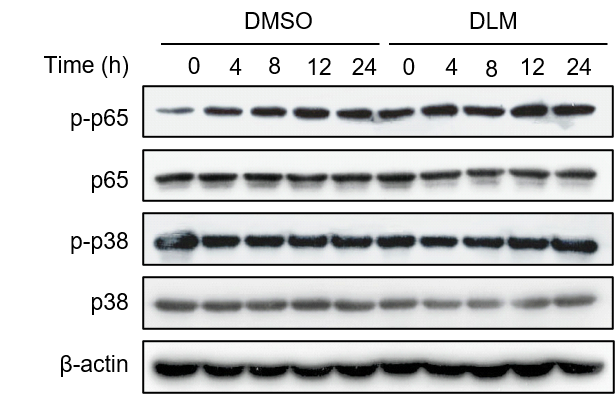

Supplement: Supplementary Figure 7 — Effect of DLM on NF-κB and MAPK pathway in macrophages. U937 cells were treated with DLM or DMSO, respectively, for 0-24 h. Western blot analysis was performed by using antibodies to p-p65, p65, p-p38, and p38, respectively. β-actin was used as control. [file Image_7.tif]

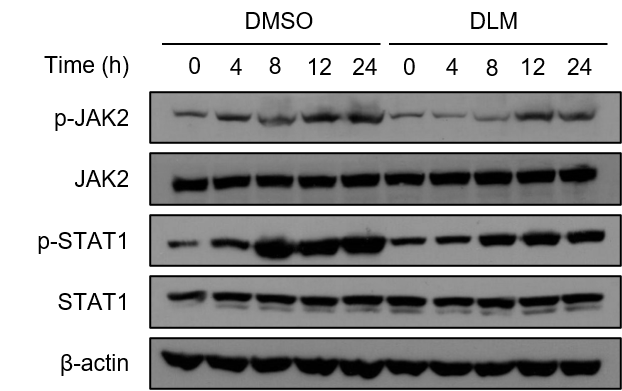

Supplement: Supplementary Figure 8 — Effect of DLM on JAK/STAT1 pathway in THP-1 cells. THP-1 cells were treated with DLM, DMSO, respectively, for 0-24 h. Western blot analysis was performed by using antibodies to p-JAK2, JAK2, p-STAT1, STAT1, respectively. β-actin was used as control. [file Image_8.tif]
